# Characterizing Challenges and Opportunities in Diagnostic Disclosure Communication in Dementia Care: A Qualitative Study of Clinicians

**DOI:** 10.1002/alz.083688

**Published:** 2025-01-09

**Authors:** Joanna Paladino, Heily Chavez Granados, Deborah Blacker, Ana‐Maria Vranceanu, Vicki Jackson, Christine S Ritchie

**Affiliations:** ^1^ Massachusetts General Hospital, Harvard Medical School, Boston, MA USA; ^2^ Massachusetts General Hospital, Boston, MA USA; ^3^ Harvard University, Boston, MA USA; ^4^ Department of Epidemiology, Harvard T. H. Chan School of Public Health, Boston, MA USA; ^5^ The Mongan Institute and Division of Palliative Care and Geriatric Medicine. Massachusetts General Hospital, Boston, MA USA

## Abstract

**Background:**

Dementia is a life‐changing condition for patients and caregivers. Response to a diagnosis often includes grief, shock, and despair. Unfortunately, evidence demonstrates inadequate use of person‐centered communication practices during diagnostic disclosure, which adds to psychological distress. Structured, person‐centered communication interventions are needed to improve the disclosure process. Our aim was to characterize communication challenges and opportunities in diagnostic disclosure.

**Method:**

Semi‐structured interviews of clinicians at 3 academic institutions, including one federally qualified health center. An interdisciplinary research team used rapid thematic analysis.

**Results:**

18 clinicians participated (MDs/NPs/SWs/RNs) across neurology, geriatrics, geriatric psychiatry, primary care. Preliminary themes include: (1) An experience of trauma frequently accompanies dementia diagnostic disclosure. Participants described devastation or denial experienced by patients and caregivers after disclosure (especially an ‘abrupt’ disclosure’), which contributed to patient threats of suicide, clinicians getting fired, or reluctance to seek care. (2) Communicating a dementia diagnosis differs from other medical conditions due to the attribution of symptoms to other causes; perception that there is ‘no fighting back’ (which may change with anti‐amyloid therapies); and the importance of early involvement of caregivers due to variation in patient cognitive capacity. (3) An incremental team‐based communication process is perceived to improve patient and caregiver experience, including: establishing therapeutic alliance; assessing awareness of the cognitive condition and personal beliefs; managing expectations and acknowledging uncertainty; sharing a clear diagnosis with emotional support; creating partnership for anticipatory guidance and planning. (4) System barriers interfere with communication, including long wait times for specialists, lack of training, and unclear roles

**Conclusions:**

A structured, psychologically‐informed communication framework for inter‐professional teams may enhance the quality and reliability of the diagnostic disclosure communication process in dementia care. Future research to develop efficacious diagnostic disclosure communication interventions has the potential to improve patient, caregiver, and clinician experience.

